# “I lost so much more than my partner” – Bereaved partners’ grief experiences following suicide or physician-assisted dying in case of a mental disorder

**DOI:** 10.1186/s12888-022-04098-5

**Published:** 2022-07-07

**Authors:** M. C. Snijdewind, J. de Keijser, G. Casteelen, P. A. Boelen, G. E. Smid

**Affiliations:** 1 ARQ Centrum’45, Nienoord 5, 1112 XE, Diemen, The Netherlands; 2 Department of Ethics, Law and Humanities, Amsterdam UMC, Academic Medical Centre, P.O. Box 22660, 1100 DD Amsterdam, The Netherlands; 3 Department of Clinical Psychology and Experimental Psychopathology, Faculty of Behavioral and Social Sciences, University of Groningen, Grote Kruisstraat 2/1, 9712 TS Groningen, The Netherlands; 4 Expertisecentrum Euthanasie, P.O. Box 13480, 2501 EL Den Haag, The Netherlands; 5 ARQ National Psychotrauma Centre, Nienoord 5, 1112 XE, Diemen, The Netherlands; 6 Department of Clinical Psychology, Faculty of Social Sciences, Utrecht University, P.O. Box 80140, 3508 TC Utrecht, The Netherlands; 7 Department of Humanistic Chaplaincy Studies, University of Humanistic Studies, Kromme Nieuwegracht 29, 3512 HD Utrecht, the Netherlands

**Keywords:** Grief, Suicide, Physician-assisted dying, Mental health, Death taboo

## Abstract

**Background:**

There is a lack of existing research on grief following the intentional death of people suffering from a mental disorder. Our study aims to provide insight into grief experiences and social reactions of bereaved persons who lost their life partners, who were suffering from a mental disorder, to physician-assisted dying (PAD) or suicide.

**Methods:**

For this mixed-methods research, we conducted a survey and in-depth interviews with 27 persons living in the Netherlands and bereaved by the death of their life partners. The deceased life partners suffered from a mental disorder and had died by physician-assisted dying (n = 12) or suicide (n = 15). Interviews explored grief experiences and social reactions. In the survey we compared self-reported grief reactions of partners bereaved by suicide and PAD using the Grief Experience Questionnaire.

**Results:**

Compared to suicide, physician-assisted dying was associated with less severe grief experiences of the bereaved partners. Participants reported that others rarely understood the suffering of their deceased partners and sometimes expected them to justify their partners’ death. Following physician-assisted dying, the fact that the partner’s euthanasia request was granted, helped others understand that the deceased person’s mental suffering had been unbearable and irremediable. Whereas, following suicide, the involvement of the bereaved partners was sometimes the focus of judicial inquiry, especially, if the partner had been present during the death.

**Conclusion:**

When individuals suffering from a mental disorder die by suicide or PAD, their bereaved partners may experience a lack of understanding from others. Although both ways of dying are considered unnatural, their implications for bereaved partners vary considerably. We propose looking beyond the dichotomy of PAD versus suicide when studying grief following the intentional death of people suffering from a mental disorder, and considering other important aspects, such as expectedness of the death, suffering during it, and partners’ presence during the death.

## Background

Grief, following the loss of a loved one, is influenced by multiple factors, including the circumstances of the death and the bereaved person’s involvement in it [1]. Death from unnatural causes, e.g., suicide, is associated with more severe and prolonged grief compared to that from natural causes [2]. Previous research including people bereaved by suicide indicated that feelings of responsibility, guilt, and rejection, and experiencing stigma are common [2–4]. Grief is more severe in people bereaved by suicide than those by other forms of sudden death [4].

Worldwide, around 1.3% of all deaths are caused by suicide [5]. In the Netherlands, 1.1% of deaths were caused by suicide in 2020 [6] and in 56.8% of such deaths, suicide was motivated by a mental disorder [7]. Worldwide, mental disorders account for a majority of suicides. A meta-analysis regarding psychological autopsies of people who committed suicide showed that 87.3% of these people had mental disorders [8]. However, a review of psychological autopsy studies found that between 5.5% and 66.7% of suicides occurred in the apparent absence of a mental health condition [9]. In line with the Dutch statistics, the 2018 US Surveillance for Violent Deaths [10] reported that circumstances were identified in 88.3% of suicides, and among them mental health problem was the most common circumstance, with 49.7% of decedents having had a diagnosed mental health problem. Unlike other forms of unnatural death – for example, accident, natural disaster, terrorism – suicide is to some extent intentional and takes perseverance to complete. Death of a loved one by suicide may be anticipated to some degree, based on previous suicide attempts or conversations, as illustrated by studies on people receiving de-medicalized assistance in suicide [11], e.g., people passing away through self-ingesting self-collected lethal medication. Previous research indicates that when the bereaved anticipate the suicide and understand the reasons behind it, they search less for explanations and have less preoccupation with it [12]. Suicide may be further divided into violent, i.e., mutilating (e.g., strangulation or high impact collision) or non-violent, i.e., non-mutilating (e.g., physician-assisted dying (PAD), drug overdose, ingestion of a deadly substance, helium inhalation). Violent deaths are more likely to generate distressing intrusive memories in the bereaved than nonviolent deaths [13].

Suicide, however, is not the only cause of death which depends on an intentional act by the person who dies; this is also the case in PAD. In this case, the death of the patient can be anticipated, as the date of death is set in advance. PAD is legally regulated in several countries worldwide [14]. In some of these countries, including the Netherlands, legal criteria of PAD include the medical condition of mental disorder of the patient. Granting such a request is still rare – while in 2021 a total of 7666 people died by PAD in the Netherlands (4.5% of total deaths), only 115 of these (1.5% of all reported PAD-cases) concerned PAD in psychiatric patients (0.07% of total deaths) [15].

Research on the impact of PAD on the mental health of bereaved relatives showed that they experienced feeling of isolation and fear of social stigma [16–19]. However, if they knew that the passing was an autonomous choice, they could accept the death and showed less symptoms of grief and posttraumatic stress disorder [16–19]. However, research on the impact of PAD on the mental health of bereaved relatives is scarce. Moreover, to the best of our knowledge, no former study focused specifically on grief following PAD in the case of a mental disorder. Therefore, we aimed to provide insight into the experience of losing a partner to PAD or suicide in case of a mental disorder. For this, we used mixed methods to conduct an exploratory qualitative semi-structured in-depth interview study combined with a comparative quantitative survey. We hypothesized that the experience of grief would differ between partners of people dying from suicide and PAD and that intensity of grief would be higher in case of suicide.

## Methods

This research was performed in accordance with relevant guidelines and regulations. We consulted the Medical Ethics Research Committee Utrecht (protocol number 19/596), who exempted the study from formal review because the Medical Research Involving Human Subjects Act (WMO) does not apply to the study. Each participant provided written informed consent before enrolling in the study.

### Recruitment and selection

The inclusion criteria for the participants were: (1) loss of a life partner to PAD or suicide in case of a mental disorder for which the decedent received treatment, or had been on a waiting list, for at least two years; (2) time since the loss was between six months and 10 years.

The required number of study participants was guided by the expected number of interviews needed to reach qualitative data saturation. Given the heterogeneity in our study population, we anticipated a larger sample would be needed to achieve this [20]. Since we also wanted to conduct quantitative comparative analyses, we aimed to include at least ten participants each in bereaved by PAD and ten bereaved by suicide of their partners. Participants were recruited through Expertisecentrum Euthanasie (EE, Euthanasia Expertise Centre, the Netherlands). While PAD related to a mental disorder is still rare, most of these cases are performed by physicians working for EE – in 2021 a total of 115 cases of PAD based on mental disorders were reported, of which 83 by physicians working for EE [15].

The EE provided information regarding all cases of PAD in patients with a mental disorder, performed by EE-physicians where a life partner had been involved. If contact information of these life partners was available, a letter was sent informing them about the research, including a response card, which could be sent to the researchers to indicate willingness to participate. One reminder was sent. Twenty-one potential participants were informed and ten positive responses were received; all met the inclusion criteria. Next, we used social media to recruit participants. An invitation was placed on the website of ARQ Centrum’45 and the University for Humanistic Studies, the Netherlands. In addition, 113 Zelfmoordpreventie (113 Suicide Prevention) tweeted about the research. Moreover, during a symposium for people bereaved through suicide, held on December 11^th^ 2020, the attendees were informed about the research and the possibility to participate. A newsletter of the Vrienden van Expertisecentrum Euthanasie contained information about the research and a call to participate. Through all these, 24 people responded, of which ten were eligible. We also used snowball sampling to recruit participants, by sending out flyers about the research in our professional network and actively asking people if they knew someone who would be eligible to participate. Thus, nine people responded, of which seven met the inclusion criteria.

### Study design

The study combined a quantitative survey with a qualitative interview. After receiving contact information of people willing to participate, a researcher called or emailed to check if they met the inclusion criteria and if so, appointments were made for the interviews. Two weeks prior to the interview, the respondent received the survey questionnaire by mail. The survey contained background and loss-related questions, the Grief Experiences Questionnaire, and a list of potential stressful life events, experienced after the loss of the partner.

The Grief Experience Questionnaire (GEQ) [21] was used to measure grief reactions. The GEQ is designed to measure two types of grief: 1) expected in any bereavement, 2) specific to suicide. The original 55-item GEQ [21] has been psychometrically evaluated in several studies [12, 22–24]. Kõlves et al. [23] performed confirmatory factor analysis [23] to evaluate the original GEQ 11 subscale structure proposed by Barrett and Scott [21] and found that eight original subscales had good reliability. We used these eight validated subscales assessing somatic reactions, search for explanation, loss of support, stigmatization, guilt, responsibility, shame, and rejection, each consisting of five items [23]. Items were rated on 5-point Likert scales (1 = *never* to 5 = *almost always*), with total scores ranging between 5 and 25. We used the translation by Wojtkowiak et al. [12]. Earlier studies established satisfactory internal [12, 22] and test-retest reliability [24]. Internal reliability (Cronbach’s alpha) in the current sample was 0.96.

The interviews were conducted by a researcher with extensive experience in this. Since the interviews were semi-structured, a topic list was used (Table 1). In the interviews, participants described the period of time leading up to the death of their partner and the time afterwards, up to their current situation. Most interviews were conducted face-to-face at participants’ homes. However due to COVID-19-related restrictions, three interviews were conducted through video calls. All participants were informed about the purpose of the study. They provided either written or oral informed consent. Interviews lasted between 46 and 231 minutes and were recorded. All interviews were held between February 2020 and March 2021.

**Table 1:** Topic list (translated, original in Dutch)

*Interview introduction*	Information about the research project Recording and confidentiality Questions Informed consent
*Introductory questions*	Reason(s) to participate Name of the partner General information about partner
*Mental disorder of the deceased partner*	First experience with partner’s mental disorder(s) and impact Good day/period, bad day/period Awareness/reaction social environment
*Care providers*	(Professional) care related to the mental disorder(s)
*Potential/possible death of the partner*	Possible death: awareness/topic of discussion/ suicidal thoughts or tendencies Conversations about ending one’s life with care provider
*Passing of the partner and life afterwards*	Way of dying, meaning for bereaved partner Farewell Period following death Reactions social environment Openness and sharing, support, loneliness
*Current situation*	Interview experience Memories Emotions Influence loss on current life Difficulties Support and comfort Openness social environment View on death Outlook on future
*Ending*	Recap Other important experiences related to the loss?

### Statistical Analyses

Analyses were performed using SPSS version 27 for Windows. Missing scale item responses were present in a mean of 0.5% of responses per case (range, 0 to 3.6%) and were handled using mean imputation. Prior to the final analyses, we checked normality by verifying that none of the variables had skewness or kurtosis values smaller than -3 or larger than 3. Descriptive analyses of the sample included frequencies and percentages for binary and categorical variables, respectively, and means and standard deviations for continuous variables. Chi-square tests and independent t-tests were conducted to examine sociodemographic and mental health related differences between the participants bereaved by PAD and suicide of their partners. Fisher’s exact tests were performed in addition to chi-square tests if expected counts were < 5 (since the significance levels did not differ, they were not reported separately). For multiple regression analyses, binary variables were dummy coded as follows: yes=1, no=0. Regression analyses evaluated the impact of the mode of partner’s death and time since death on mental health related variables, with and without adjustment for the effects of other loss-related variables, specifically, being present during the partner’s death and violent mode of partner’s suicide. For this, independent variables were entered into the regression in two steps, and the significance of the R^2^ change was calculated for the second step. Visual inspection of residual plots confirmed homoscedasticity. There was no evidence of multicollinearity, as indicated by tolerance values > 0.25. Alpha level was set at .05 for statistical significance.

### Interview Analyses

All recorded interviews were transcribed. By inductive coding, we conducted a thematic analysis using ATLAS.ti. The first three interviews were open coded by three researchers and these codes were extensively compared and discussed until an agreement was reached. Based on this, a coding scheme was drafted that was further developed through coding of the next seven interviews. Thereafter, codes were frequently grouped and regrouped into overarching themes. Codes were added when new information emerged during analyses. Data analysis was frequently discussed between the authors and agreement was reached by discussing the interpretation of specific quotations in the context of the entire interview. Intersubjectivity was assured by coding and discussing three interviews with two other researchers and discussing excerpts, codes, and interpretations of other interviews with another researcher.

## Results

### Sample characteristics

Twenty-seven bereaved participants were included in this study, of whom twelve had experienced the death of their partner by PAD and fifteen by suicide. Using data from the interviews, the cause of death was further divided into violent, i.e., mutilating (e.g., strangulation or high impact collision) or non-violent, i.e., non-mutilating (e.g., PAD, drug overdose, ingestion of a deadly substance, helium inhalation). Fifteen participants were present during the death. Being present during the partner’s suicide only occurred in non-violent suicides. Table 2 lists the characteristics of the participants and the mode of death of their partners as well as the GEQ-scores.

**Table 2:** Sociodemographic variables, stressful life events, and grief experiences by mode of partner’s death

	Full sample		Cause of partner’s death					
	(N = 27)		Suicide (N = 15)		PAD (N = 12)			
	N	%	N	%	N	%	χ^2^ (df = 1)	
Gender								
Female	13	48.1	10	66.7	3	25.0	4.64	*
Male	14	51.9	5	33.3	9	75.0		
Higher education^1^	17	63.0	14	93.3	3	25.0	13.35	***
Children^2^	20	74.1	12	80.0	8	66.7	0.62	
Filed a complaint^3^	2	7.4	2	13.3	0	0.0	1.73	
Present during the partner’s death^4^	15	55.6	3	20.0	12	100.0	17.28	***
Violent partner’s death^5^	8	29.6	8	53.3	0	0.0	9.10	**
Stressful life events after partner’s death								
Experienced illness, injury	1	3.7	1	6.7	0	0.0	0.83	
Family member ill, injured	6	22.2	5	33.3	1	8.3	2.41	
Parent, child or sibling died	4	14.8	4	26.7	0	0.0	3.76	
Friend or other family member died	2	7.4	0	0.0	2	16.7	2.70	
Breakup of relationship	8	29.6	8	53.3	0	0.0	9.09	**
Relationship problem	8	29.6	6	40.0	2	16.7	1.74	
Job loss or inability to find a job	2	7.4	1	6.7	1	8.3	0.03	
Fired	0	0.0	0	0.0	0	0.0	-	
Financial problem	1	3.7	1	6.7	0	0.0	0.83	
Contact with the law	1	3.7	1	6.7	0	0.0	0.83	
Theft or loss of valuables	2	7.4	1	6.7	1	8.3	0.03	
	M	SD	M	SD	M	SD	t (df = 25)	
Age	61.02	13.11	54.27	11.45	69.46	9.99	-3.62	**
Years since death	2.63	2.52	3.52	2.81	1.51	1.59	2.21	*
Age partner	58.15	14.61	49.83	11.03	68.56	11.71	-4.27	***
Duration of relationship	30.00	18.22	21.20	14.81	42.04	15.58	-3.55	**
Number of stressful life events	1.30	1.20	1.87	1.25	0.58	0.67	3.21	*
Grief Experience Questionnaire								
Somatic reactions	9.04	3.95	10.67	3.77	7.00	3.25	2.67	*
Search for explanation	11.35	3.93	12.85	3.27	9.48	4.01	2.41	*
Loss of support	10.17	4.38	12.43	4.07	7.33	2.93	3.65	**
Stigmatization	9.63	4.82	11.93	5.20	6.75	2.05	3.25	**
Guilt	9.87	4.07	11.20	4.44	8.21	2.93	2.00	
Responsibility	7.72	3.53	9.20	4.05	5.88	1.38	2.71	*
Shame	8.04	3.55	9.40	3.79	6.33	2.39	2.44	*
Rejection	9.05	4.32	10.68	4.59	7.00	3.02	2.39	*

^1^ vs. lower education

^2^ vs. no children

^3^ vs. not filed a complaint

^4^ vs. not present during the partner’s death

^5^ vs non-violent death

* *p* < .05; ** *p* < .01; *** *p* < .001

As shown in Table 2, participants whose partners died by suicide were significantly higher educated and more often female compared to those whose partners died by PAD. In addition, they – and their partners – were significantly younger, and the duration of the relationship was shorter. At the moment of study, more time had passed for the participants whose partners died by suicide. Stressful life experiences following the partner’s death did not differ significantly between the two groups except the experience of breakup in a relationship, which occurred more often in the suicide- than the PAD-bereaved group. Mean GEQ scores were lower for the PAD-bereaved group on all subscales. Between-group differences in mean GEQ subscale scores were all significant except for the guilt subscale.

### Unnatural death

Both suicide and PAD are considered non-natural causes of death. Because of this, the body of the deceased needs to be released following inspection by the medical examiner. Respondents bereaved by PAD mentioned that this was just a formality. Often, the medical examiner was informed by the physician about the upcoming PAD beforehand and the examination did not take much time.

*It was all taken care of by the physician of the euthanasia association [Expertisecentrum Euthanasie]. A medical examiner had to come, had to be called, or the public prosecutor or something like that. That was all taken care of, I was not involved in that. I don’t know if there was a medical examiner, or that she called the public prosecutor.* (r.19, PAD, present during the death)

Respondents bereaved by suicide mentioned that besides a medical examiner, police officers were present to investigate the circumstances of the death. Several respondents were questioned about their involvement in the suicide and were not allowed near their partner’s body during investigation. This weighed heavily on them. An extensive examination involving the bereaved respondents seemed to occur more often in non-violent suicides where the partner had been present. However, one of our interviewees mentioned that sometimes a non-violent suicide could almost pass for a natural death – in which no medical examination or police investigation would take place.

*She [the physician] was about to declare a natural death. Had I only put the note [of the partner, stating she ended her life herself] somewhere else. But then again, if the medical examiner had come and he would’ve had a suspicion and those bottles were still in the kitchen... Should I have thrown them out? No, that only raises suspicion. You shouldn’t do that.* (r.18, non-violent suicide, present during the death)

Following PAD, contact with the officials was organized by the physician, who was also the liable person. In contrast, following suicide, the respondent was often the one who needed to initiate contact with the officials. How and when this should take place was not always self-evident. The respondents faced questions, such as when should they report their partner missing and how long should they wait to inform officials to be certain that their partner actually died. Though sometimes they did not recognize these moments of choice until later.

*We had the deal that if something happened, we wouldn’t try to save each other. We didn’t want to end up in a persistent vegetative state. Afterwards I felt regret, I went to the neighbors too soon. Should I not have waited longer? ( … ) I begged them, twice, three times. First to the officer on duty, to the police, to the officers on duty, each time there was a higher ranking... if they would please stop the resuscitation, but they just kept going. They just kept going.* (r21, violent suicide, not present during the death)

### Social environment’s reactions to the partner’s mental disorder

The respondents mentioned that their social environment – friends, family, co-workers, or acquaintances – was often not fully aware of the decedent’s mental disorder and its impact. They explained this by saying that their partner preferred other people not to know, since they wanted to appear as normal and healthy as possible and not to be treated differently, or because they were ashamed of the condition. Others feared an unsupportive response from outsiders, based on past experiences. The common experience was that other people, even loved ones, did not notice the severity of the disorder and its impact. This might explain why the social environment often did not understand the intention of the deceased to end his or her life. It resulted in openly questioning this intention and sometimes trying to convince to not follow through, or blaming the respondent afterwards for not stopping the partner. Respondents had to face negative emotions and opinions of others instead of feeling supported by them. While these reactions were not limited to suicide, the granting of a PAD request sometimes made it easier for the respondents to explain the severity of the disorder and the suffering of their partner to others; it seemed to validate the intention to end one’s life.

*Anyone may know. If I talk about his death – when I’m somewhere new, or have new colleagues – I tell them my husband died not so long ago, he had so many complaints, it was no longer doable, he received euthanasia. That I’ll tell them right after.*

*Interviewer (I): And people’s reaction … ?*

*Then it must have been bad. I mean, you don’t receive euthanasia just like that, do you?* (r17, PAD, present during the death)

Respondents often received supportive reactions concerning the loss of their partner. The reactions concerning the fact their partners had intentionally ended their lives varied from wondering how this could have happened and feeling that it should have been prevented, to recognizing the tragic situation for which there had been no alternative solution. Respondents felt they were sometimes blamed for the suicide of their partner, while respondents whose partner died by PAD did not feel this way. Being blamed left respondents feeling vulnerable, isolated and angry, and resulted in loss of friends and family.

*And then [their reaction] ‘oh, suicide?’. It is still taboo. ‘Was he happy?’ I think [partner] was very happy here, but at certain moment … You can’t explain it. Then I’ll leave it. Never mind, because people are so short sighted. ‘Was there no help? Hasn’t there been this [or that]?’ Yeah, as if we hadn’t tried everything ourselves. ( … ) It’s not only suicide, that is a taboo, but also just dealing with grief.* (r11, non-violent suicide, not present during the death)

*They [the partner’s parents] blame me for everything, they blame me for the death of* [partner] (r21, violent suicide, not present during the death)

*His brother turned me in as a suspect. ( … ) His brother wanted my role in this story to be re-examined. So, everything had to be handed in [to the police]: phones, laptops, whatever. What a suspicion, on top of everything that’s already happened.* (r27, PAD, present during the death)

Some respondents, from both groups, found it important to be open about the way their partner had died, motivated by a wish to contribute to a society where these topics were talked about more openly.

Many respondents advocated that physicians and psychiatrists should be more willing to consider and grant a request for PAD in patients with mental disorders. This was often mentioned as one of the reasons to participate in the research.

### Questions and doubts

Some respondents indicated that they began to doubt themselves after the death of their life partners. Self-doubt concerned their choice of partner, but also their own current feelings and thoughts. These questions and insecurities were mostly present in respondents who had not foreseen the death of their partner.

*I thought, that’s not right, how can this be? How could this happen, that I’m feeling happy again? My partner died half a year ago. How can I feel happy again, that’s strange? I almost felt ashamed because of it. ( … ) I felt ashamed, I thought I’m doing something weird, I’m weird, I’m the only one. I’m already the only one that this had happened to, and now I’m also the only one who feels this way. That makes you judge yourself. ( … ) When I’m passionate about something … then I just get on with it. And right now, I just can’t. And then I start doubting myself. Am I right? Am I on the right track? Then I’m overcome by doubt and with that my self-confidence [declines] ( … ) It’s not like it was before, I’m not so free anymore, that’s a part of grief.* (r20, violent suicide, not present during the death)

Respondents who were aware of the impending death, did not have self-doubt or unanswered questions, as they were convinced that their partners willingly chose to die and at the time, it was the only option for their partner to find relief.

*Well, you obviously prefer to continue [living] as you’re used to. However, you know that’s not on option anymore, it’s no longer there. And well, I don’t really have a hard time dealing with it. ( … ) I can’t think of anything that I find difficult or have problems with. No, I knew what the situation was and that it would never change and that I would have to continue like this.* (r13, PAD, present during the death)

### Mode of death predicting grief experiences and stressful life events

Table 3 shows the factors associated with GEQ scores, as well as the number of stressful life events the participants endured since their partners’ death. In the first step of the multiple regression analyses, dying by PAD was found to be associated with less somatic reactions, search for explanation, loss of support, stigmatization, guilt, responsibility, and lower number of stressful life events. In the second step, we added the predictors violent death and being present during the death. Violent death was associated with increased somatic reactions, loss of support, stigmatization, guilt, and responsibility of the participants. PAD was associated with increased (i.e., decreased loss of) support. The number of stressful life events was associated with longer time since the death and violent death.

**Table 3:** Multiple regressions predicting grief experiences and stressful life events based on mode of partner’s death and time since death

	Step 1								Step 2										
	B	95% CI		Beta		F		R^2^	B	95% CI		Beta		F		R^2^	ΔR^2^	ΔF	
Somatic reactions						3.65	*	0.23						4.18	*	0.43	0.20	3.84	*
Years since death	-0.19	-0.82	0.44	-0.12					-0.19	-0.77	0.38	-0.12							
Physician-assisted dying^1^	-4.04	-7.19	-0.90	-0.52	*				-2.83	-7.40	1.75	-0.36							
Violent death^2^									5.29	1.18	9.40	0.62	*						
Present during the death^3^									2.00	-3.15	7.14	0.26							
Search for explanation						4.05	*	0.25						2.50		0.31	0.06	0.97	
Years since death	-0.43	-1.05	0.19	-0.28					-0.42	-1.05	0.21	-0.27							
Physician-assisted dying^1^	-4.24	-7.33	-1.15	-0.55	**				-2.59	-7.61	2.42	-0.33							
Violent death^2^									2.34	-2.16	6.85	0.28							
Present during the death^3^									-0.46	-6.10	5.18	-0.06							
Loss of support						6.42	**	0.35						6.21	**	0.53	0.18	4.27	*
Years since death	-0.07	-0.72	0.58	-0.04					-0.10	-0.68	0.48	-0.06							
Physician-assisted dying^1^	-5.24	-8.45	-2.02	-0.60	**				-5.58	-10.20	-0.97	-0.65	*						
Violent death^2^									5.83	1.69	9.98	0.62	**						
Present during the death^3^									4.25	-0.95	9.44	0.49							
Stigmatization						5.14	*	0.30						4.81	**	0.47	0.17	3.44	*
Years since death	0.12	-0.61	0.86	0.06					0.11	-0.57	0.79	0.06							
Physician-assisted dying^1^	-4.94	-8.61	-1.27	-0.52	*				-4.31	-9.72	1.11	-0.45							
Violent death^2^									6.10	1.24	10.96	0.59	*						
Present during the death^3^									3.24	-2.85	9.33	0.34							
Guilt						2.02		0.14						5.71	**	0.51	0.37	8.19	**
Years since death	-0.13	-0.82	0.56	-0.08					-0.08	-0.63	0.47	-0.05							
Physician-assisted dying^1^	-3.26	-6.68	0.17	-0.41					1.67	-2.72	6.05	0.21							
Violent death^2^									5.14	1.20	9.08	0.59	*						
Present during the death^3^									-2.60	-7.53	2.33	-0.32							
Responsibility						3.53	*	0.23						5.68	**	0.51	0.28	6.28	**
Years since death	0.02	-0.55	0.58	0.01					0.05	-0.43	0.53	0.04							
Physician-assisted dying^1^	-3.29	-6.12	-0.47	-0.47	*				0.34	-3.47	4.16	0.05							
Violent death^2^									4.05	0.63	7.47	0.53	*						
Present during the death^3^									-1.75	-6.04	2.53	-0.25							
Shame						3.16		0.21						2.07		0.27	0.07	0.99	
Years since death	-0.20	-0.78	0.38	-0.14					-0.22	-0.80	0.37	-0.15							
Physician-assisted dying^1^	-3.47	-6.34	-0.60	-0.49	*				-3.89	-8.54	0.76	-0.56							
Violent death^2^									2.80	-1.38	6.97	0.37							
Present during the death^3^									2.35	-2.88	7.58	0.34							
Rejection						2.90		0.19						1.69		0.24	0.04	0.58	
Years since death	-0.17	-0.88	0.54	-0.10					-0.18	-0.91	0.55	-0.11							
Physician-assisted dying^1^	-4.03	-7.56	-0.51	-0.47	*				-3.80	-9.61	2.01	-0.45							
Violent death^2^									2.70	-2.52	7.92	0.29							
Present during the death^3^									1.49	-5.05	8.02	0.17							
Number of stressful life events						7.68	**	0.39						6.10	**	0.53	0.14	3.14	
Years since death	0.16	-0.01	0.34	0.34					0.16	0.00	0.32	0.34	*						
Physician-assisted dying^1^	-0.95	-1.81	-0.10	-0.40	*				-0.67	-1.94	0.61	-0.28							
Violent death^2^									1.34	0.20	2.48	0.52	*						
Present during the death^3^									0.53	-0.90	1.96	0.22							

^1^ vs. suicide

^2^ vs. non-violent death

^3^ vs. not present

* *p* < .05; ** *p* < .01; *** *p* < .001

## Discussion

The aim of this study was to provide insight into grief experiences following the loss of a partner, suffering from mental disorder, to PAD or suicide. Our results show that people bereaved by suicide and PAD of their partners not only lose their loved one, but also experience a lack of understanding and support from others. Although both PAD and suicide are considered unnatural causes of death, their implications for bereaved partners vary considerably. Following PAD, all persons involved are supported by the physician, who initiates and handles all contacts with officials. In contrast, following suicide, the bereaved partner has to find out what to do if he or she was present during the time of death or has found the partner’s body. Furthermore, many people bereaved by suicide at some point had to make difficult on-the-spot decisions, regarding their partner, to which they felt ill-prepared.

### A good death?

A good death may be seen from the perspective of its impact on grief, mental health, and wellbeing of the bereaved. People bereaved by suicide are at higher risk of developing mental health issues and suicidal behavior compared to those bereaved by other modes of death [25]. Notably, it has been shown that loss to violent death is strongly associated with difficulty in accepting the loss, and consequently, with other prolonged grief symptoms (e.g., a continued sense of shock, bitterness, emptiness, and yearning), symptoms of posttraumatic stress disorder, and depression [26]. Given the predictive effects of prolonged grief on reduced mental health over time [27], it is likely that mental health issues in people bereaved through suicide are in part grief related. The analysis of GEQ showed that PAD had a protective effect on the severity of the grief experiences of bereaved partners compared to suicide. It is possible that the protective effect of PAD may be relevant to mental health issues and suicidal behavior of people bereaved by PAD. Future research is needed to further investigate this association.

**Fig. 1:**
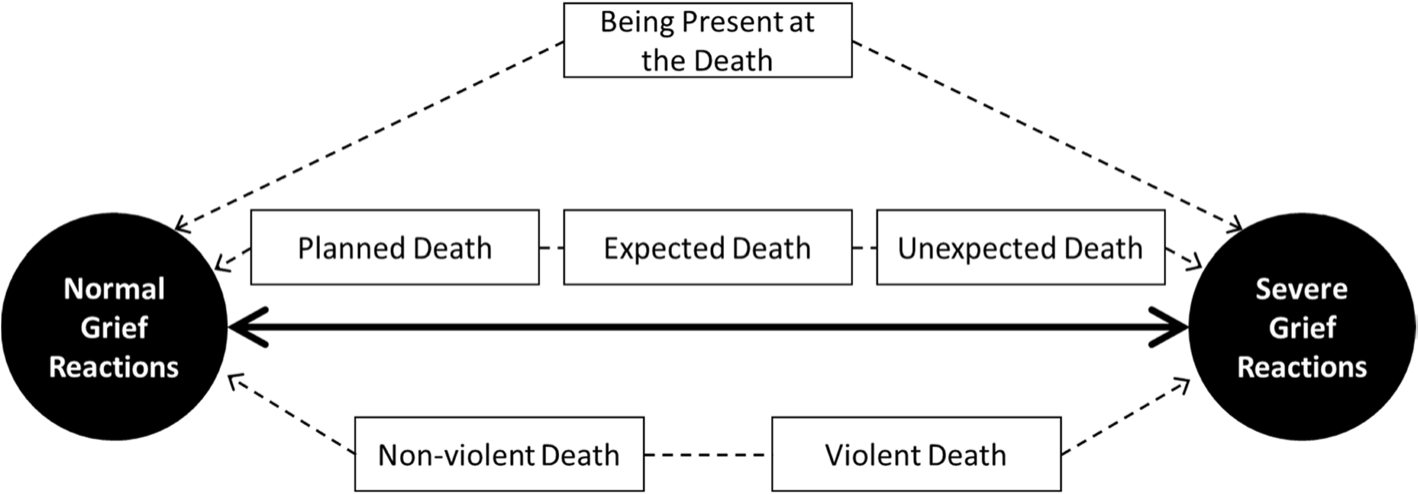
A heuristic, hypothetical model of death-related factors influencing bereaved partners’ grief experiences following suicide and PAD related to a mental disorder

Addition of other independent variables showed that it might be worthwhile to look beyond the PAD-suicide dichotomy. A partner’s violent death impacted the grief experiences of the bereaved in addition to the suicide. The interviews offered an even more nuanced view into grief than did the GEQ results. In addition to the cause of death (PAD or suicide), bereaved partners’ grief experiences were influenced by the death being violent or non-violent, irrespective of whether they were present during the death, and anticipated it. A previous study into grief experiences of people bereaved by suicide showed that the more the loved one’s suicide was expected, the less the bereaved seek explanations and meaning after the death [12]. Further similar studies might reveal a pattern in which grief experiences in the context of suicide and PAD might be placed on a scale from the experience of a good death without severe grief, to one of a horrible death followed by severe grief. PAD would be on the one end of this scale, and an unexpected and violent suicide on the other. In between we will find a planned non-violent suicide with the partner present, a planned non-violent suicide without the partner present, and an unexpected non-violent suicide without the partner present. Figure 1 illustrates this hypothesis. Overlapping this spectrum are reactions from outsiders who show little understanding of the severity of the mental disorder. Unlike suicide, following PAD, the bereaved partner can counter these reactions by the fact that a physician came to the conclusion that the suffering of the patient was unbearable and without a prospect of improvement. This can not only convince outsiders, but also reinforce the bereaved partner’s own interpretation of the past situation. Feeling isolated and experiencing a lack of understanding is also reflected in the ‘loss of support’ and ‘stigmatization’ subscales of the GEQ. A previous interview study on stigma suggested that death taboo still exists in Western society, more specifically concerning sudden deaths [28]. The authors reasoned that this might be related to the “shocking or unusual nature; causing others significant unease” [28]. Our study findings suggest that stigmatization may be less prevalent after PAD, despite the unnatural cause of the death. Thus, death taboo might be less related to the unnatural cause of death, but more to the violent and unexpected manner of it.

We have seen that bereaved persons sometimes struggle with questions and doubts following the loss of their significant others. Although the subscale ‘search for explanation’ did not show significant outcomes, the interviews showed that some people were confronted with questions – most often when the suicide was unexpected. The subscales ‘guilt’ and ‘responsibility’ also seem to be associated with these inner questions and doubts. Being confronted with blame and having the feeling of not being understood by the social environment seemed to stir up inner doubts even more.

Our findings show that feelings of self-doubt following the partner’s death seem to be related to the death being unanticipated. When the death was expected, some of the questions (e.g., about why and how) had been resolved by talking to the partner – questions that bereaved partners following an unexpected death were still struggling with. Anticipating the death of the partner and having conversations about it are parts of a process known as anticipatory grief [29]. Conversations about the death seem to strengthen the bond between partners. However, they also confront the healthy partner with the past, present, and future losses [29], as losses are not limited to the death of the partner but may include altered relationships or other social and economic changes. In this context it is interesting to note that a program focused on family connections in people with suicidal behavior disorder [30] may contribute to reducing grief associated with the mental illness of a loved one, and it would be worthwhile to investigate the effects of the family connection program on grief if the loved one eventually dies by suicide.

In our sample, participants bereaved by PAD were in the relationship with their partners longer than those bereaved by suicide. In addition to the process leading to PAD, that may support anticipatory grief, the longer duration of the relationship may have helped anticipate the death.

It was evident from the study that bereaved partners lost so much more than their partner. Timely conversations may help them prepare for these losses in advance. Concepts of preparedness and anticipatory grief might be helpful to open such conversation between care providers. In case of planned suicides, specifically, in jurisdictions that do not allow PAD related to a mental disorder, care providers need to be aware of the challenges of partners having discussions with each other, and try to prevent the other partner from being seen as aiding and abetting the suicide.

### Study strengths and limitations

This is the first study on grief experiences of life partners of people who died by PAD or suicide in case of a mental disorder. By including partners of people who died by mutilating or non-mutilating suicide, we obtained a nuanced view on grief of losing a loved one who intentionally ended his or her life due to a mental disorder. By combining a survey and an interview, we were able to show the broad variety of experiences and provide an indication of the severity of the grief.

The study had the following limitations. First, for the quantitative analyses, our sample size was small. Although a small sample size is generally associated with a low power to detect statistically significant associations, we found several significant associations to confirm our hypotheses, suggesting that our sample size yielded enough statistical power. However, generalizability of our findings may be limited by the small sample size. In addition, generalizability may be limited due to self-selection of participants in the study. It could bias the results if people were motivated to participate because of negative experiences, thus overrepresenting the severity of mental health problems. Conversely, if people were motivated by a desire to advocate for a specific method to die (particularly expected in PAD), this could lead to underrepresentation of severe mental health problems. Since the actual experiences of the participants were not one-sided – they also mentioned difficulties experienced in the process of PAD and positive aspects of suicide – we expected that self-selection bias did not skew our results.

It is noteworthy that our study took place in the Netherlands, and the results may not be generalizable to other jurisdictions.

We found gender differences between participants bereaved by suicide and PAD. It is known that more women die by PAD and more men by suicide [31]. Given the predominance of heterosexual relationships, gender distribution in our sample reflects expected general population patterns, with participants bereaved by suicide and PAD being most often women and men, respectively. We also found mean age differences between the two groups. Studies of granted euthanasia requests due to mental disorders found that most patients were under treatment for over ten years [32]. Thus, it is possible that people who died by suicide in our sample had a shorter mean duration of treatment compared with people who died by PAD. In addition, the time required to grant a request for PAD may be a contributing factor. Patients are likely to direct their initial request for PAD to their treating physician. If the physician refuses, the patient may turn to the EE. For psychiatric patients, the mean waiting time at the EE has been 10 months during the past years [32]. In addition, to meet the due care criteria of irremediable suffering, remaining treatment options sometimes need to be tried before considering PAD as an option.

Due to the COVID-19 pandemic, three interviews were conducted through video calls. The impact of COVID-19 was addressed during the interviews. Social restrictions related to COVID-19 may have impacted the experience of grief of the participants, such as recent feelings of isolation, or a decline in social contacts. Some respondents thought the measures, mainly social distancing, indeed contributed to these feelings.

### Implications

Pending future studies with larger samples, our study provides initial evidence that PAD due to mental disorder may be associated with an increased understanding of the extent of the suffering of the deceased person by the bereaved and their social environment. In addition, the findings provide a more nuanced view on suicide, which is often considered a traumatic, violent, and sudden death. Our results show that it might do more justice to the act and its experience to distinguish between ways of suicide, so that non-sudden and non-violent suicides are not overlooked. In practice, non-violent suicide death cases might be reported as a natural deaths, thus limiting the reliability of current registrations. Specifically, planned suicides may be underreported. However, further research is needed on this.

According to the guideline of the Dutch Association for Psychiatry (NVvP), the physician considering PAD in case of a mental disorder should involve significant others of the patients in the process towards PAD and provide care to them afterwards [33]. Our study results support this recommendation. In addition, the results suggest that involvement of significant others should include attention for their perspective on a good death and their grief in order to further reduce the risk of prolonged grief and other negative grief experiences.

## Data Availability

The datasets generated and/or analysed during the current study are not publicly available, due to the ethically sensitive nature of the research and potential participant identifiers in the datasets. Selected data are available from the corresponding author on reasonable request.

## Abbreviations

EE: Expertisecentrum Euthanasie (Euthanasia Expertise Centre, The Netherlands)
GEQ: Grief Experience Questionnaire
PAD: Physician-assisted dying
